# Developing a feasible and sensitive judgement bias task in dairy cows

**DOI:** 10.1007/s10071-021-01563-8

**Published:** 2021-10-11

**Authors:** Louise Kremer, Cornelis G. van Reenen, Bas Engel, Eddie A. M. Bokkers, Sabine K. Schnabel, Jozef T. N. van der Werf, Laura E. Webb

**Affiliations:** 1 Animal Production Systems Group, Wageningen University and Research, Wageningen, The Netherlands; 2 Wageningen Livestock Research, Wageningen University and Research, Wageningen, The Netherlands; 3 Biometris, Wageningen University, Wageningen University and Research, Wageningen, The Netherlands

**Keywords:** Judgement bias, Punisher, Affective state, Feasibility, Sensitivity, Cows

## Abstract

Judgement bias tasks (JBTs) are used to assess the influence of farm practices on livestock affective states. The tasks must be adjusted to the species and age group of focus. In cattle, most JBTs were designed for calves instead of adult cows. This study aimed to develop a JBT suitable for adult dairy cows, combining feasibility, validity, sensitivity and repeatability. Three JBTs were developed in which cows were trained to reach or avoid reaching a feeder, the location of which signalled a reward or punisher. The tasks differed in terms of punisher—cows being allocated either to “no-reward”, an air puff or an electric shock. Cows were then exposed twice to three ambiguous positions of the feeder, on two separate occasions. Speed of learning and proportions of correct responses to the conditioned locations were used to assess the feasibility of the task. Adjusted latencies to reach the ambiguous feeder positions were used to examine whether response patterns matched the linear and monotonic graded pattern expected in a valid and sensitive JBT at baseline. Latencies to reach the feeders in the two repeated testing sessions were compared to assess ambiguity loss over tasks’ repetitions. The validity of using spatial JBTs for dairy cows was demonstrated. While the effect on JBT feasibility was nuanced, the punisher did influence JBT sensitivity. None of the JBTs’ repeatability could be supported. We conclude that using an air puf as punisher led to the most sensitive, yet non-repeatable, JBT for dairy cows.

## Introduction

During their life, dairy cows typically experience a number of potentially challenging events, such as overcrowding (Fustini et al. 2017) or regrouping (Phillips and Rind 2001), which are likely to influence their welfare. Animal welfare is defined here as a multidimensional concept that revolves around three major areas: the animal’s ability to display natural behaviour, its physical condition and its affective state (Hemsworth et al. 2015; Blokhuis et al. 2019). An accurate assessment of cow welfare must, therefore, take into account affective states (Watanabe 2007). Affective states relate to individual positive and negative mental states (Duncan 2006; Fraser 2009) and encompass both emotions and moods. We define emotions here as “states elicited by rewards and punishments” (Rolls 2000), and moods as background states resulting from the accumulation of emotions (Mendl et al. 2010b). In animal welfare studies, the Judgement Bias Task (JBT) has commonly been used to objectively assess affective states (for meta-reviews, see Lagisz et al. 2020 and Neville et al. 2020). The JBT is assumed to open a window into the affective states of animals by studying “judgement biases”, i.e. the influence of affective states on the interpretation of ambiguous stimuli (Eysenck et al. 1991; Mendl et al. 2009; Roelofs et al. 2016). Like humans (e.g. Blanchette and Richards 2010), animals in positive affective states are more likely to interpret ambiguous information more positively, hence to be more optimistic, than animals in more negative states—and vice versa (Harding et al. 2004). In livestock research, JBTs are generally used to investigate the impact of supposedly negative (e.g. shearing: Sanger et al. 2011) or positive husbandry practices (e.g. human grooming: Baciadonna et al. 2016) on the affective states of farm animals (Baciadonna and McElligott 2015). To this day, however, the application of JBT in dairy cows remains anecdotal (one study only: Crump et al. 2021), and our understanding of dairy cows’ emotional life is consequently limited. By proposing and designing a JBT for dairy cows, dairy scientists may become more inclined to implement this unique tool in their research—an important step to further develop our knowledge of dairy cows’ affective states, and eventually meet the dairy industry’s ambition for improved animal welfare (Weary and Von Keyserlingk 2017).

In practice, judgement bias is assessed by investigating whether an individual displays a behaviour associated with the anticipation of a relatively positive or negative outcome in response to ambiguous situations. To measure judgement bias, researchers typically train animals to discriminate between two conditioned cues signalling either a reward, which represents the positive cue (P), or a less positive reward or punisher, which represents the negative cue (N). These conditioned cues typically differ according to a unique sensory continuum (auditory: e.g. Brilot et al. 2010, tactile: e.g. Brydges et al. 2011, visual: e.g. Bateson and Matheson 2007). Two main types of JBT exist—namely the active choice task and the Go/NoGo task. In active choice tasks, animals learn to display one active response to P (e.g. touch a circle symbol) and an alternate active response to N (e.g. touch a triangle symbol). In Go/NoGo tasks, animals are trained to perform one active response to P (i.e. “Go”) and to suppress this active response in response to N (i.e. “NoGo”). Following the lead of previous studies conducted on herbivores (sheep: e.g. Doyle et al. 2010a,b, calves: e.g. Lecorps et al. 2018, goats: e.g. Baciadonna et al. 2016, horses: e.g. Briefer Freymond et al. 2014), this paper focused on a spatial discrimination task based on a Go/NoGo paradigm. Once trained, animals are generally exposed to three ambiguous cues, one at the midpoint of the sensory scale between P and N (A), one halfway between A and P (Ap) and one halfway between A and N (An) (Lagisz et al. 2020). Eventually, the judgement bias in Go/NoGo tasks is assessed based on the proportions of Go responses to, or latencies to reach, the ambiguous cues—relatively high proportions of Go responses and short latencies reflecting more optimistic judgements, hence more positive affective states. Judgement bias, therefore, simply provides a relative measure of affective states (Bateson and Nettle 2015) and JBTs can only be used to make comparative inferences of affective states either between different populations or different treatments (Lagisz et al. 2020). JBTs remain, nonetheless, the only tool to date allowing researchers to investigate both positive and negative shifts in animal affective states—which explains its popularity and widespread use within the scientific community.

When designing a JBT, researchers must take various practical and theoretical considerations into account (Baciadonna and McElligott 2015; Bethell 2015; Roelofs et al. 2016; Hintze et al. 2018; Neville et al. 2020).

First, the *feasibility* of the task must be ensured to facilitate the adoption and the implementation of the JBT within different research groups. In practice, the feasibility of the JBTs is challenged by the duration of the training period and the number of successfully trained animals within this period (e.g. Roelofs et al. 2016; Hintze et al. 2018). Animals are typically considered trained once they reach a pre-determined training criterion—which may in some cases demand a high number of training sessions. Even with extensive training, some animals may still be excluded from the experiment for not meeting the training criterion rapidly enough (e.g. Jones et al. 2017). Consequently, the JBT results may be biased toward a population of “learners”, which may limit the generalisation of the findings (Roelofs et al. 2016). Strategies to optimise the training procedures are, therefore, warranted to enhance what we call here the tasks’ feasibility—particularly in experimentations involving large animals like dairy cows, where handling is challenging (Douphrate et al. 2009).

Second, the *internal validity* of a JBT must be guaranteed to ensure a correct interpretation of the results (Mendl et al. 2009; Hintze et al. 2018). The internal validity of a tool is defined as the strength of causality between a treatment and a measured outcome (Slack and Draugalis 2001). In the JBT, this relates to the extent to which animal responses are caused by the exposure to ambiguous situations. In a valid JBT, the baseline responses (i.e. before application of any treatment, hence under reference conditions) should follow a monotonic graded pattern: latencies to reach the cues should increase as the ambiguous cues are further away from P on the sensory scale. This pattern of responses ensures that individuals respond to the ambiguous cues within the framework of the JBT and according to the learnt outcomes of the conditioned cues (Roelofs et al. 2016; Hintze et al. 2018). In other words, an erratic pattern of responses to the ambiguous cues suggests that the animals consider the middle cues as novel or meaningless rather than ambiguous, and therefore that the animals do not rely on the learnt positive and negative outcomes associated with P and N to make their decisions to approach or not the middle cues (Mendl et al. 2009; Gygax 2014; Jones et al. 2017; Hintze et al. 2018). The internal validity of a JBT should be ensured at baseline, before using the task to investigate the effects of certain treatments on animal affective states.

**Fig. 1:**
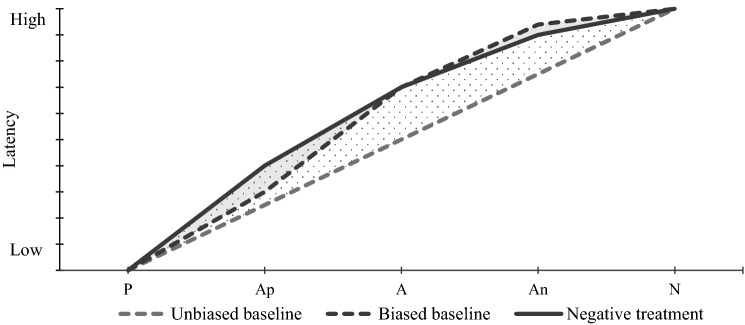
Example of expected latencies in response to the conditioned and ambiguous cues before (dotted lines) and after (solid line) the application of a negative treatment in the context of a Judgement Bias Task (JBT). Treatments inducing negative shifts in animal affective states lead to more pessimistic responses to the ambiguous cues, i.e. the latencies to reach the ambiguous cues will be higher. The linear monotonic graded baseline represents the pattern of latencies obtained in response to the cues before the application of any affective treatment in the context of a valid and sensitive JBT (grey dotted line). In a less sensitive JBT (black dotted line), the profile of responses before the application of the treatment is not linear. The differences in responses obtained before and after the negative treatment are greater in the case of an unbiased baseline (dotted area) compared with a negatively biased baseline (grey area). In the negatively biased scenario, the treatment-induced negative affective shift may not be detected—or only in response to the ambiguous positive Ap cue. *P* positive, *Ap* ambiguous positive, *A* truly ambiguous, *An* ambiguous negative, *N* negative. Microsoft Powerpoint was used to create the artwork

Third, the *sensitivity* of the task should be maximised to ensure the identification of treatment-induced shifts in animal affective states. The sensitivity of a tool is defined as the tool’s ability to detect the effect it measures. In a JBT, sensitivity relates to the task’s ability to detect both positive and negative treatment-induced judgement biases. In a sensitive JBT, the baseline response pattern of latencies across the ambiguous cues should ideally be linear. JBTs with baseline patterns biased toward N, for instance, are likely to be less sensitive to treatment-induced negative affect, because negative judgement biases may then only be detectable at the ambiguous cues positioned closest to P (Fig. 1), as suggested elsewhere (Mendl et al. 2009; Lagisz et al. 2020). In practice, JBT sensitivity seems highly heterogeneous (Lagisz et al. 2020; Neville et al. 2020). Several factors inherent to the JBT set-up have recently been identified as sensitivity modulators (Lagisz et al. 2020)—including the training reinforcement combination (e.g. large reward/small reward, or reward/punisher) and the sensory continuum selected for the cues (e.g. spatial or auditory). Researchers should, therefore, carefully consider these methodological aspects when designing JBT. Failure to account for these modulators increases the risk of false negatives—i.e. the JBT fails to detect the effect of a treatment on animal affective states (e.g. Horváth et al. 2016). Such type II errors may lead researchers to erroneously claim that certain husbandry practices do not affect livestock welfare while these are, in fact, beneficial or detrimental to the animals. Designing a JBT with a set-up that maximises the task’ sensitivity is also all the more valuable when considering a study population of females like dairy cows—as females appear to be less sensitive than males to judgment bias (Lagisz et al. 2020).

Fourth, the *repeatability* of the task should also be ensured to avoid erroneous interpretation of the results (Roelofs et al. 2016). Here, we define repeatability as the task’s ability to ensure that repeated exposures to the ambiguous cues do not lead to ambiguity loss. If the animals associate a specific outcome with the ambiguous cues over several exposures, then the ambiguous cues become, by definition, no longer ambiguous (Roelofs et al. 2016; Hintze et al. 2018). In practice, repeated exposures to ambiguous cues have been associated with increased reluctance to approach the cues (Doyle et al. 2010b) – which could falsely be interpreted as a treatment-induced pessimistic bias within the context of a longitudinal study. Before using any newly developed JBT, researchers must hence assess the repeatability of their task at baseline to ensure its suitability for longitudinal designs. The necessity to develop repeatable JBTs arises from recent evidence demonstrating the importance of endogenous factors, such as personality traits, on animal responses to the JBT (e.g. fearfulness in calves, Lecorps et al. 2018). Unlike trans-sectional studies, longitudinal studies allow researchers to control for individual differences that would otherwise bias the outcomes of the JBT. Longitudinal studies, furthermore, allow for the introduction of extra training sessions between the first testing session (baseline before the application of the treatment) and the second testing session (after the application of the treatment). These additional sessions may serve as a “wash-out” period—potentially reducing the likelihood of animals remembering their first encounter with the ambiguous cues (Doyle et al. 2010b). The ability of such wash-out period to potentiate ambiguous loss over repeated testing remains, nonetheless, to be proven.

**Fig. 2:**
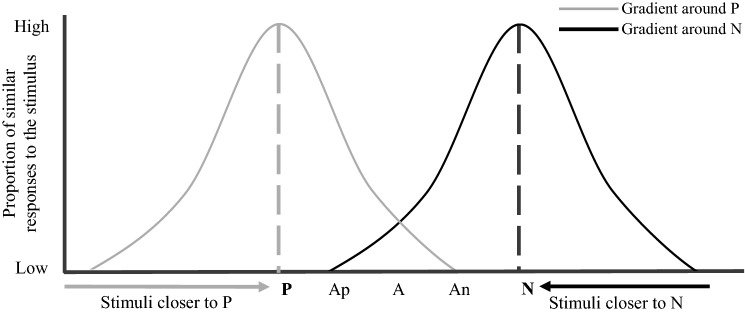
Generalisation gradients represented as Gaussian distributions around the negative cue N in dark grey and around the positive cue P in light grey (adapted from Roelofs et al. 2016). Animals are more likely to display the same behaviour learnt in response to the conditioned cue (e.g. P or N) when faced with unconditioned cues similar to the conditioned one. *Ap* ambiguous positive cue, *A* truly ambiguous cue, *An* ambiguous negative cue. Microsoft PowerPoint was used to create the artwork

In light of the aforementioned considerations, our goal was to develop a feasible, valid, sensitive and repeatable JBT for dairy cows. In this paper, we focused on a specific methodological aspect of the JBT: the combination of reinforcers. The combination of reinforcers can modulate the feasibility of a discrimination learning task, by influencing an animal’s ability to discriminate between two perceptually similar conditioned cues. For example, in a visual discrimination task using sucrose as a positive reinforcer, bees’ visual discrimination of two shades of the same colour was enhanced by the use of a quinine solution instead of water as the negative reinforcer (Avarguès-Weber et al. 2010). The combination of reinforcers can also impact the sensitivity of JBTs (Mendl et al. 2009; Roelofs et al. 2016). Animal decision-making about whether or not to approach an ambiguous cue is thought to result from the interaction of the two generalisation gradients around the positive and the negative conditioned cues (Fig. 2) (Roelofs et al. 2016). The generalisation gradient describes the phenomenon by which individuals transfers a learnt behavioural response from one conditioned cue to other perceptually similar cues (Guttman and Kalish 1956; Schechtman et al. 2010) and depends on the inherent properties of the reinforcer associated with the stimulus. In humans, for instance, threat-intensity has been shown to widen the generalisation gradient around N (Dunsmoor et al. 2017). Modifying the combination of reinforcers by replacing the type of punisher may, thus, influence JBTs in terms of both feasibility and sensitivity.

Consequently, we developed three JBTs differing solely in terms of negative reinforcer, hereafter called punishers—using either a “no-reward” (i.e. the absence of feed delivery), an air puff or an electrical shock. The “no-reward” and air puff punishers were selected based on previous studies that successfully developed JBTs using feed-reward/“no-reward” (e.g. Hintze et al. 2018; Crump et al. 2021) and feed-reward/air puff combinations as reinforcers in herbivores (e.g. Destrez et al. 2013; Lecorps et al. 2018). The electric shock was selected based on its proven efficacy to contain cattle on pasture via electric fencing (McDonald et al. 1981) and its common use as a punisher in rodents (e.g. Enkel et al. 2010). The overall validity of our spatial discrimination task was assessed to investigate whether cows effectively perceived the intermediary spatial cues as ambiguous. We assessed the repeatability of each JBT separately to investigate their potential for longitudinal studies. The repeatability of the tasks was evaluated while implementing a wash-out period of two weeks between two testing periods. Finally, we completed our assessment of each JBT by comparing the tasks in terms of feasibility and sensitivity. We hypothesised that, in dairy cows: (1) The reward/punisher combination influences the feasibility of the JBT; (2) Spatial JBT is overall valid in dairy cows; (3) The reward/punisher combination influences the sensitivity of the task; and (4) All JBTs are repeatable when a wash-out period is implemented between two testing periods, regardless of the combination of reinforcers applied.

## Materials and methods

The study was conducted between May and October 2018 at the experimental farm of Dairy Campus, Leeuwarden, The Netherlands. All procedures complied with the Dutch law for animal experiments and were approved by Wageningen University Committee on Animal Care and Use.

### Animals and management conditions

Experimental animals were mid-lactating *Friesian x Holstein* dairy cows (*N*=39; 3.7 ± 0.1 years old on average when enrolled in the study, 23.1 ± 0.8 kg of milk per day; 692.8 ± 11.1 kg body weight two weeks before the start of the study) between their first and third lactation. The study was divided into two experimental batches of three months each (N1=21 and N2=18, respectively). Focal cows (i.e. cows used in the experiment) were housed in a solid floored free-stall barn, opened to the exterior. Focal cows were housed with 32 ± 3 (mean ± sd) companion cows (i.e. cows not used in the experiment) that were mixed and replaced according to the farm’s regular schedule and need. Dim artificial lighting was provided 24 h/24 h. Cows had access to four automatic brushes and 54 flexible cubicles with gel mattresses (AgriProm) overed with sawdust. Cows received a total mixed ration of grass silage (10.5 % of dry matter), maize silage (15.8 %), brewer’s grains (4.5 %), grinded whole soy (7.7 %), grinded whole wheat (8.1 %), concentrates (14.5 %) and minerals (1.8 %) around 9:00 h-that was pushed towards the fences around 17:00 h. Additionally, cows had free access to four automatic concentrate dispensers delivering a pre-set daily amount of concentrates based on individual milk production and *ad libitum* access to four water troughs. Milking occurred twice a day between 08:00–9:00 h and 15:00–16:00 h. To facilitate handling, the barn was divided into two pens during workdays (Mon–Fri) and focal cows were separated from their companions. The three punisher treatments were balanced for focal cows’ parity.

### Experimental design

Focal cows were subjected to one of three judgement bias procedures that differed in terms of punishers. The punisher was either an inaccessible feed-reward coupled with a 10s time-out (NOTH), a 5 bar air puff (AIR) (SPECAIR HI 275/35), or an electric shock (ELEC) (GARMIN Delta XC, 7/18). The feed-reward, which consisted of 150 g of concentrates, remained out of cows’ reach and sight by storing it inside the receptacle of a wood-crafted feeder on wheels. The air puff was delivered via an air pipe connected to the bottom of the feeder bowl - where cows would usually eat the feed when the latter was made accessible. The air puff experience also included hearing a loud noise, as a result of sudden air release. The electric shock was delivered from a neck collar. Punishers were assumed to initially induce frustration (NOTH), fear and frustration (AIR), and a combination of pain, fear and frustration (ELEC). Assumptions of punisher-induced affective states were based on the appraisal theories, which postulate that specific situations trigger specific affective states (Sander et al. 2005). Since frustration is thought to emerge from a situational inability to attain a goal, we hypothesised that all punishers elicited frustration because cows could not fulfil their desire to eat, a desire likely triggered by the smell of the concentrates emanating from the receptacle. Additionally, we hypothesised that the AIR- and ELEC-punishers elicited fear because these stimuli of low intrinsic pleasantness were sudden, unfamiliar and unpredictable (Désiré et al. 2004). Arguably, however, the release of the air puff and of the electric shock may have become more predictable across repeated exposures. Finally, we assume that the ELEC-punisher induced pain in cows.

A pilot study was also conducted before the main experiment, during which cows were trained to reach a bucket filled with 150 g of concentrates during 7 consecutive trials per day for 2 weeks. The objectives of this pilot study were to ensure that (1) cows were willing) to eat 1.050 kg of concentrates on top of their daily ration of concentrates, (2) cows were willing to participate in the task over an extended period and (3) to optimise cow handling inside the experimental facility. In this way, the experimenters ensured that cows would not stop responding to P across training sessions due to a lack of interest in the reward and they learnt to handle cows in a stress-free and efficient manner.

**Fig. 3:**
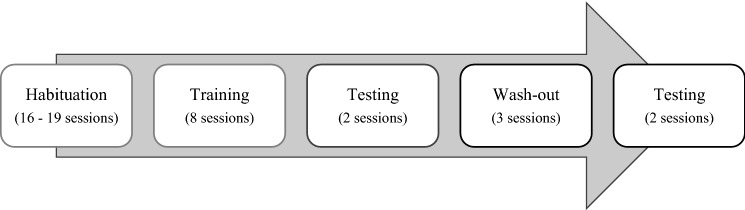
Timeline of the phases of the judgement bias task for one experimental batch. All sessions took place during the weekdays (Mon–Fri). All groups were habituated on the same days. One to two habituation sessions were conducted per day. Habituation sessions lasted 5–15 min per cow. Extra-habituation sessions were provided to cows who did not reach the habituation criterion after 16 sessions. Extra sessions were provided within 24 h following the 16th habituation session. During training, three groups of three cows could be trained per day (i.e. two days were needed to train all cows in one batch to the same training session). In the first batch, a seventh group was also trained in the evenings after milking. Training sessions lasted 30–45 min per cow. Two consecutive days were required to complete one testing session with all cows. Testing sessions also lasted 30–45 min per cow. The same holds for wash-out sessions*.* Microsoft PowerPoint was used to create the artwork

Two experimenters remained present during the study—experimenter 1 (L.K) being in charge of preparing the experimental facility and releasing the appropriate reward or punisher, and experimenter 2 being in charge of handling the cows. Experimenter 2 differed between the two batches, while experimenter 1 remained the same throughout. The judgement bias procedures consisted of several phases (Fig. 3) described in detail below.

### Judgement bias

#### Experimental facility

**Fig. 4:**
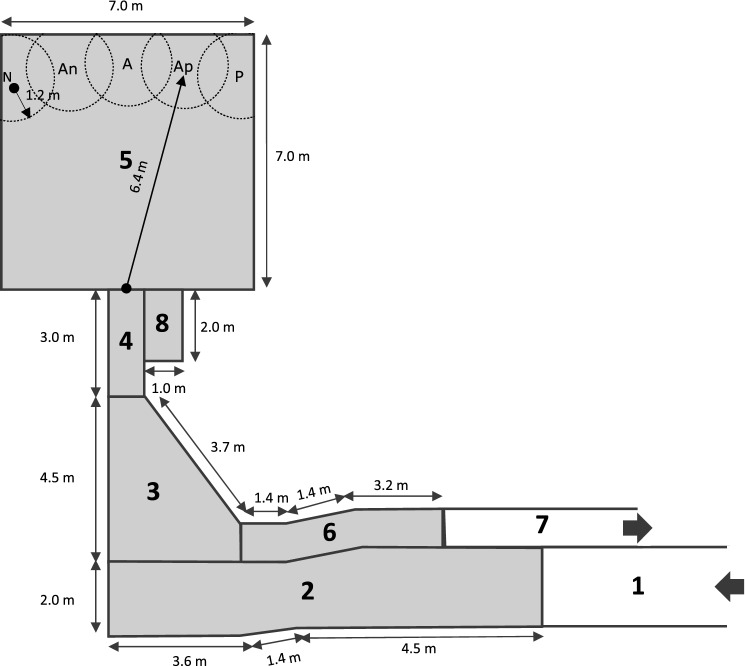
Layout of the experimental facilities used for the judgement bias procedures*.* 1: Entry corridor. It belongs to the milking corridor and is used to bring the groups of cows from the home-pen to the waiting area*.* 2: Waiting area. Cows remained here while a cow from their group was habituated, trained or tested in the arena*.* 3: Turning area. Cows were used to turn on themselves inside the turning area to re-enter the arena in-between two consecutive trials. In the meanwhile, experimenter 1 positioned the feeder at the correct location for the next trial, and out of sight from the cow*.* 4: Starting box. Cows remained in the starting box for 15 s before each trial. A metal bar was positioned behind the cow to prevent her from going backward. The metal bar was lifted up shortly before the saloon door was opened*.* 5: Testing arena. The feeder was always presented at one of the 5 indicated locations (N/An/A/Ap/P) during a training or a testing trial. P and N locations were balanced across punishers*.* 6: Resting area. Cows were released onto the resting area at the end of a session while a cow from their group was trained or tested inside the arena*.* 7: Exit corridor. All cows from a group were thereafter brought back together to their home-pen via the exit corridor*.* 8: Experimenters’ office. Experimenters could observe the cows inside the testing arena via a screen connected to the cameras located inside the testing arena. The experimenters also had access to an automatic console, that they used to release concentrate and air puff by distance. The remote control to deliver the electric shock was also placed in the experimenters’ office*.* Microsoft PowerPoint was used to create the artwork

The judgement bias procedures took place in a dedicated 7×7 m and 3.5 m high wooden-walled arena located in-between the barn and the milking parlour. The arena had a roof, a concrete floor and no window. Artificial light (4000 K cool white) was provided with six fluorescent tubes and two spotlights. Four cameras (CAMCOLBUL2, Velleman, Belgium) were installed inside the testing arena. A starting box adjacent to the arena allowed the cows to enter the testing arena. Cows were brought from their home-pen to the arena in groups of three (test group) using a familiar milking route (Fig. 4). The order of test groups was randomly determined each day, except for replacement group 7, initially trained during the evenings.

#### Habituation

**Fig. 5:**
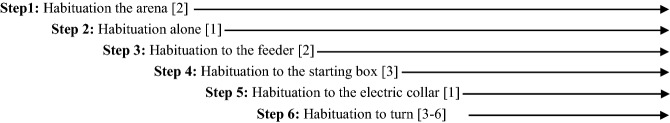
The six steps of habituation to the judgement bias task. The numbers in square brackets refer to the number of habituation sessions given per step of habituation*.* Microsoft PowerPoint was used to create the artwork

The habituation procedures took place in six incremental steps (Fig. 5). The order of habituation (first, second and third cow) within a group was determined based on the punisher allocation: each of the six possible sequences of three punishers was randomly allocated to at least one test group per batch. This order was maintained during the entire experiment. During step 1, the habituation session consisted in a unique 5 min trial during which cows were habituated to remain in groups of three inside the arena. Three buckets filled with 150 g of concentrates were initially interspersed in the arena-as an incentive for cows to explore the arena. From step 2, cows were habituated to stay 90 s alone inside the arena, with two buckets filled with concentrates positioned in diagonal corners. From step 3, the buckets were replaced by two automatic feeders that were always positioned in a concentric fashion from the centre of the arena. An electric wire was connected between the feeder and a console located in the experimenter’s office so that experimenter 1 could release 150 g of concentrates each time a cow would successfully reach the feeder. If the cow did not reach the feeder within 90 s, concentrates were still released. If the cow still did not reach the feeder on her own 30 s after the release of concentrates, experimenter 1 entered the arena and gently orientated the cow toward the feeder while encouraging her vocally and petting her hips. From step 4, cows were habituated to being inside the starting box for 15 s before the start of each trial. From step 5, cows were habituated to wearing the electrical collar - that was gently put on the cow’s neck in the waiting area prior to the habituation session of the first test cow. All cows were habituated to wearing the collar, regardless of their allocated punisher. From step 6, each session consisted of 2 trials, in-between which cows were trained to turning around in the turning area to re-enter the arena. At this point, cows only had access to one feeder positioned in a pre-selected corner of the arena, balanced across groups. If the cow did not reach the feeder within 90 s, concentrates were still released. If the cow still did not reach the feeder on her own 30 s after the release of concentrates, experimenter 1 entered the arena and gently orientated the cow toward the feeder while encouraging her vocally and petting her hips. Cows were considered habituated to the judgement bias procedures once they had reached the feeder for two consecutive trials within 90 s.

#### Training

Once habituated, cows were subjected to eight training sessions. Cows were brought in groups of three to the waiting area, before being individually trained. During the training sessions, cows had to learn to discriminate between two feeder locations situated either on the far-right or far-left corner of the arena (N and P in Fig. 4). One location signalled a positive outcome (P) while the other signalled a negative outcome (N). P and N cues were randomly assigned to the far-left or the far-right corner of the arena per cow, and the rewarding corner was balanced across punishers to avoid side bias. For practical reasons, the rewarding corner remained the same within a test group of 3 cows. Training sessions consisted of 7 consecutive trials, of maximum 90 s each. Regardless of the trial type (N or P), the receptacle of the feeder was always filled with 150 g of concentrates to prevent cows from relying on olfactory cues to discriminate between the spatial cues. Before each trial, the focal cow remained in the starting box for 15 s, after which a saloon door leading onto the arena was opened. Experimenter 2 then firmly slapped three times with both hands on the cow’s hips to encourage her to enter the arena. If the cow did not enter the arena, three additional slaps were given. If unsuccessful, the experimenter eventually pushed the cow inside the arena. The trial would only start once the cow crossed the virtual door line with one hoof. The order of exposure to P and N was pseudo-randomly determined, so that each training session would start with a positive followed by a negative trial and would always end with a positive trial (e.g. *P–N*–N–P–N–P–*P*, where letters in italic indicate trials that remained fixed across all sessions). The cows could not be exposed more than two consecutive times to the same trial type, mostly to minimise the negative experiences following the negative trials and ensuring cow’s willingness to participate in the training. Cows were trained to display Go responses to P and to reach the feeder to get 150 g of concentrates. Experimenter 1 scored a Go response if the cow reached P within 20 s. If the cow did not reach P within 90 s, concentrates were still released. If the cow still did not reach the feeder on her own 30 s after the release of concentrates, experimenter 1 entered the arena and gently orientated the cow toward the feeder while encouraging her vocally and petting her hips. Cows were also trained to display NoGo responses to N to avoid their assigned punisher. Experimenter 1 scored a NoGo if the cow did not reach N within 90 s. When the cow reached N, AIR or ELEC punisher was immediately delivered and the cow was released from the arena. NOTH-cows remained ten additional seconds in the arena before being released, to prevent them from associating the N cue with the immediate end of the trial. The trial ended either after 90 s, or once the cow reached N -i.e. when the cow crossed the 1.2 m—radius circle around the feeder (Fig. 4). Cows were considered trained once they made at least 13 correct responses out of 14 trials during two consecutive sessions. This criterion was selected based on existing literature in farm animals, where it ranges between 80% of correct responses over 20 trials (Hintze et al. 2018) to 100% over 10 trials (Lecorps et al. 2018). All cows had exactly 8 training sessions (i.e. 56 trials), regardless of when they met the training criterion. Latencies to reach the conditioned cues during training were video recorded.

#### Testing

After eight training sessions of seven consecutive trials each, the testing phase started. One testing phase consisted of two testing sessions of seven trials conducted on two separate days. The testing phase always started with a positive and a negative trial—as a reminder of the task for the cows - and ended with a negative and a positive trial, in this order. The same procedures as those used during the training were applied for P and N cues. Cows were exposed to the ambiguous cues during three consecutive trials (3rd, 4th and 5th). The feeder, always filled with 150 g of concentrates, was positioned either in the middle of the arena (A), in-between A and P (Ap), or in-between A and N (An) at 6.40 m from the starting line (Fig. 4). The order of exposure to the ambiguous cues was based on studies conducted in sheep and calves (Destrez et al. 2012; Lecorps et al. 2018). Ambiguous cues were presented in the order Ap/An/A during the first testing session, and in the order A/Ap/An during the second testing session. The ambiguous locations were neither rewarded nor punished with an air puff or an electric shock. The ambiguous trials ended *as soon as* the cow entered the 1.2 m zone around the feeder with one front hoof. Therefore, cows did not experience a 10s time-out when they reached the ambiguous cues—unlike NOTH-cows when they reached N. Latencies to approach the cues were video recorded and scored as done during the training sessions. The same procedures as the ones used during the first testing session were applied in a second testing session, and cows were tested in the same order as they were during the first testing session. The second testing phase occurred after a wash-out period of 10 days. The wash-out period consisted of three sessions of regular training, aiming at reducing the risk of cows remembering the outcomes of the ambiguous cues. Furthermore, maintaining training until the second testing period minimised the risk of altering cow affective states between the two testing sessions. JBT training may indeed provide a form of cognitive enrichment (Roelofs et al. 2016) which could improve animal affective states (Pomerantz and Terkel 2009; Zebunke et al. 2013). Stopping animal training could, therefore, negatively influence affective states. In total, each cow was, therefore, tested 4 times - two times before and two times after the wash-out period.

### Data analyses

All statistical analyses were conducted using R version 4.0.5 (R Core Team 2020). The significance level was set at α < 0.05. The tendency level was set at α < 0.10. Data and scripts are available in a public repository DOI.4121/15125193.

#### Feasibility

The feasibility of each JBT was assessed based on cows’ responses to the conditioned cues during training. One AIR-cow was removed from the study due to aggressiveness towards the experimenters, and two cows were excluded from the analyses since their punishers (ELEC and NOTH) were mistakenly switched during the third training session. Thus, in total, thirty-six cows were included in the dataset (NOTH-cows: 12, AIR-cows: 12, ELEC-cows: 12).

Learning success was assessed based on the proportion of trained cows after 8 training sessions and cows’ learning speed for each JBT. The effect of the punisher on the proportion of trained cows was investigated using a Fischer exact test. Learning speed was the number of sessions required for each cow to reach the training criterion. Learning speed was scored as a 9 when cows did not reach the training criterionafter 8 training sessions. Differences in learning speed according to the punisher were investigated using a Friedman test and specifying Group as block.

Cow discrimination between P and N was assessed by calculating latencies to reach P and N during training. Response variables were expressed as remaining latencies, i.e. ${\text{remaining latency}} \left( {Cue_{i} } \right) = 1 - \frac{{{\text{Latency}}\left( {Cue_{i} } \right)}}{90},$ in such a way that a NoGo response corresponds to a remaining latency of zero. Remaining latencies were analysed using a generalised linear mixed model (GLMM, McCulloch et al. 2014). Analysis was by approximate maximum likelihood estimation using Laplacian integration, employing routine glmmTMB (Brooks et al. 2017). The used GLMM comprises a logit link for fixed and random effects and a beta distribution for the proportions of remaining latencies. It allowed to model NoGo responses with a probability *p* and Go responses with a probability *1-p*. For a Go response, where a non-zero proportion of remaining latencies was observed, fixed effects on the logit scale included main effects for batch, punisher (NOTH, AIR, or ELEC), cue type (P or N) and the interaction term between punisher and cue type. Random effects of the intercepts were included for sessions nested within cows nested within groups. In the NoGo part, the logit of *p* was modelled with fixed effects for punisher and cue type and random effect of the intercept for cows. The interaction term between punisher and cue type in the NoGo part was dropped from the final model because it had no significant effect on cows’ probability to display NoGo responses. Wald tests were performed to assess the fixed effects, both for the Go and NoGo parts of the model. For subsequent pairwise comparisons, based on estimated marginal means (on the logit scale), a Bonferroni correction was applied for multiple testing. As is customary, adjusted p-values higher than 1 were rounded to 1.

Each response to P and N was also scored as either correct (1) or incorrect (0), based on the conditioned cue and the latency to reach the cue. In response to P, latencies smaller or equal to 20 s were scored as 1 (based on Henry et al. 2017), while latencies above 20 s were scored 0. In response to N, a NoGo response was scored as 1, and a Go response was scored as 0. These binary data were also analysed using a GLMM employing routine glmmTMB. This specific GLMM comprised a logit link and a binomial (Bernoulli) distribution. Fixed effects on the logit scale included main effects for batch, punisher (NOTH, AIR, or ELEC), cue type (P or N) and the interaction term between punisher and cue type. Random effects included sessions nested within cows nested within groups, following the recommendation from Gygax (2014). Wald tests were performed to assess the fixed effects. Again, subsequent pairwise comparisons included a Bonferroni correction.

#### Internal validity: discrimination among the cues

The validity of our JBT was assessed based on cows’ responses to the cues during testing sessions of the first period. Analyses were conducted on cows who met the training criterion. One NOTH-cow was removed from the study due to miscarriage (*n* = 25; NOTH-cows: 6, AIR-cows: 9, ELEC-cows: 10).

Internal validity was evaluated based on cow discrimination of the cues. For each cow, latencies to reach the cues were averaged over the two testing sessions (hence not training) of the first testing period. Adjusted latencies were thereafter calculated using the following expression: ${\text{Adjusted latency}} \left( {Cue} \right) = \frac{{{\text{Latency}}\left( {Cue} \right) - {\text{Latency}}\left( {{\text{mean}}\left( P \right)} \right)}}{{{\text{Latency}}\left( {{\text{mean}}\left( N \right)} \right) - {\text{Latency}}\left( {{\text{mean}}\left( P \right)} \right)}}$. (Mendl et al. 2010a). Adjusted latencies were used to account for differences in walking speed between cows. First, the overall validity of our spatial Go/NoGo task (i.e. for all punishers combined) was assessed by investigating differences in adjusted latencies between two adjacent cues (e.g. P and Ap; An and N) using Wilcoxon signed rank tests. Second, the internal validity of each JBT was assessed separately by investing differences in adjusted latencies between the truly ambiguous cue A and the conditioned cues P and N using Wilcoxon signed rank tests.

#### Sensitivity

The sensitivity of the test was assessed by calculating the divergence of cow experimental responses from the theoretical unbiased baseline. During the first testing period, the positive area $\mathrm{A}+,$ and negative area $\mathrm{A}-$ were calculated for each cow between the curves obtained for the experimental adjusted latencies and the theoretical line of adjusted latencies (i.e., respectively, $\mathrm{A}+,$ above and $\mathrm{A}-$ below the theoretical unbiased baseline). To assess the divergence from the expected theoretical line according to the punisher, the response variable signed area *SA* was determined as follow: $SA=\mathrm{sign}(\mathrm{max}(\mathrm{A}+,\mathrm{ A}-)) \times \mathrm{ max}(\mathrm{A}+,\mathrm{ A}-).$ Negative *SA,* thus, indicates a punisher-driven positive judgement bias, while a positive *SA* indicates a punisher-driven negative judgement bias. *SA* differences according to the punisher were analysed by using Wilcoxon rank-sum tests. At cue level, differences between the adjusted latencies to reach each ambiguous cue according to the punisher were also assessed using Wilcoxon rank-sum tests.

#### Repeatability

The repeatability of each JBT was assessed based on cows’ responses to the cues during the first and the second testing periods. Analyses were conducted on cows who met the criterion established during the initial training period (*n* = 25, NOTH-cows: 6, AIR-cows: 9, ELEC-cows: 10). For each cow, latencies to reach each cue were averaged over the two testing sessions of one testing period,before calculating the respective adjusted latencies. Differences between adjusted latencies during the first and the second period were analysed using Wilcoxon signed rank test for each type of ambiguous cues separately. Spearman’s rank correlation coefficients between adjusted latencies to reach the ambiguous cues during the first and second testing periods were also calculated as a measure of JBT repeatability.

## Results

Odds ratios (OR) and associated 95 % confidence intervals (CI) are specified for binary data (including the NoGo part associated with a probability *p*); means of the raw data ± standard error and inter-quartile ranges (IQR) are given otherwise.

### Feasibility

#### Learning success

The Fisher exact test did not reveal statistical evidence for significant differences in proportion of trained cows between punishers (NOTH: 7/12, AIR: 9/12, ELEC: 10/12, *p*=0.526). Similarly, there was no statistical evidence for significant differences in learning speed between punishers (NOTH: 7.2 ± 0.63, IQR=3.3; AIR: 6.6 ± 0.64, IQR=3.5; ELEC: 5.9 ± 0.68, IQR=4.3, *p*=0.249, χ^2^=2.78, df=2).

#### Go responses to the conditioned cues

**Fig. 6:**
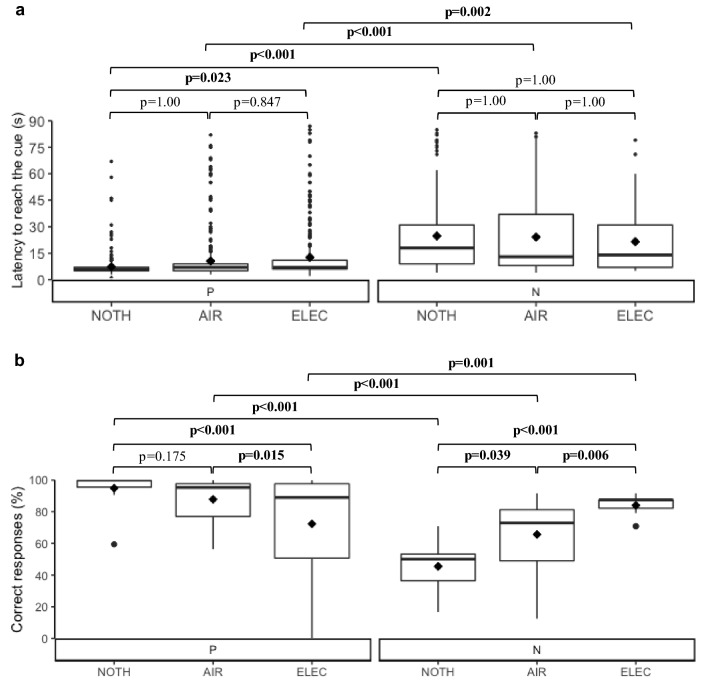
The graph depicts the box plots of (**a**) the latencies to reach the conditioned cues for Go responses and (**b**) the proportion of correct responses to the conditioned cues according to the punisher across the eight training sessions. For each box, ♦ represents the mean value. *NOTH* punisher is the absence of a reward, *AIR* punisher is an air puff, *ELEC* punisher is an electric shock, *P* positive cue, *N* negative cue. Significant *p*-values are written in bold. R 4.0.5 was used to create the artwork

There was a significant interaction effect between the punisher and the cue type on cows’ latencies to reach the cues (*p*=0.005, χ^2^=10.6, df=2). Pairwise comparisons showed no statistical evidence that NOTH-cows were significantly faster to reach P than AIR-cows (*t*=1.38, df=1954 for all pairwise comparisons) or that AIR-cows were significantly faster to reach P than ELEC-cows (*t*=1.68). However, NOTH-cows were significantly faster to reach *P* than ELEC-cows (*t*=3.02). Furthermore, there was no evidence for significant differences in latencies to reach N between the punishers (NOTH vs AIR: *t*= – 0.29, AIR vs ELEC: *t*=0.05, NOTH vs ELEC: *t*= – 0.20). Finally, latencies to P were significantly smaller than latencies to N, regardless of the punisher. Results are detailed at the punisher and the cue levels in Fig. 6a.

#### NoGo responses to the conditioned cues

There was a significant effect of the punisher on cows’ probability to display NoGo responses to the cues (*p*<0.001, χ^2^=23.0, df=2). Pairwise comparisons revealed that the probability to display NoGo responses was significantly lower for NOTH-cows compared with AIR-cows (OR=0.42, 95 % CI [0.23–0.77], *p*=0.014, *t*=–.82, df=1954 for all pairwise comparisons), and for AIR-cows compared with ELEC-cows (OR=0.24, 95% CI [0.08-0.71], *p*=0.029, *t*=.60). Additionally, the probability to display NoGo responses was significantly lower for NOTH-cows compared with ELEC-cows (OR=0.10, 95 % CI [0.05–0.19], *p*<0.001, *t*=7.23). There was also a significant effect of the cue type on cows’ probability for NoGo responses: cows were significantly less likely to display NoGo responses to P than to N (OR: 67, 95% CI [23–196], *p*<0.001, χ^2^ = 467.4, df=1, t=7.67).

#### Proportion of correct responses

There was a significant interaction between the punisher and the cue type on cows’ proportion of correct responses to the cues (*p*<0.001, χ^2^=146.3, df=2). Pairwise comparisons showed no statistical evidence that NOTH-cows displayed significantly more correct responses to P than AIR-cows (OR=2.67, 95% CI [1.17–6.07], *t*=2.34, df=1974 for all pairwise comparisons), but they revealed that AIR-cows displayed significantly more correct responses to P than ELEC-cows (OR=3.12, 95% CI [1.53–6.33], *t*=3.14). NOTH-cows also displayed significantly more correct responses to P than ELEC-cows (OR=8.31, 95% CI [3.77–18.32], *t*=5.25). Furthermore, NOTH-cows displayed significantly less correct responses to N than AIR-cows (OR=0.37, 95% CI [0.19–0.73], *t*=–.86), and AIR-cows displayed significantly less correct responses to N than ELEC-cows (OR=0.28, 95% CI [0.14–0.58], *t*= – 3.42). Likewise, NOTH-cows displayed significantly less correct responses to N than ELEC-cows (OR=0.11, 95% CI [0.05–0.22], *t*= – 6.12). Results at the cue level are detailed in Fig. 6b.

### Internal validity

**Fig. 7:**
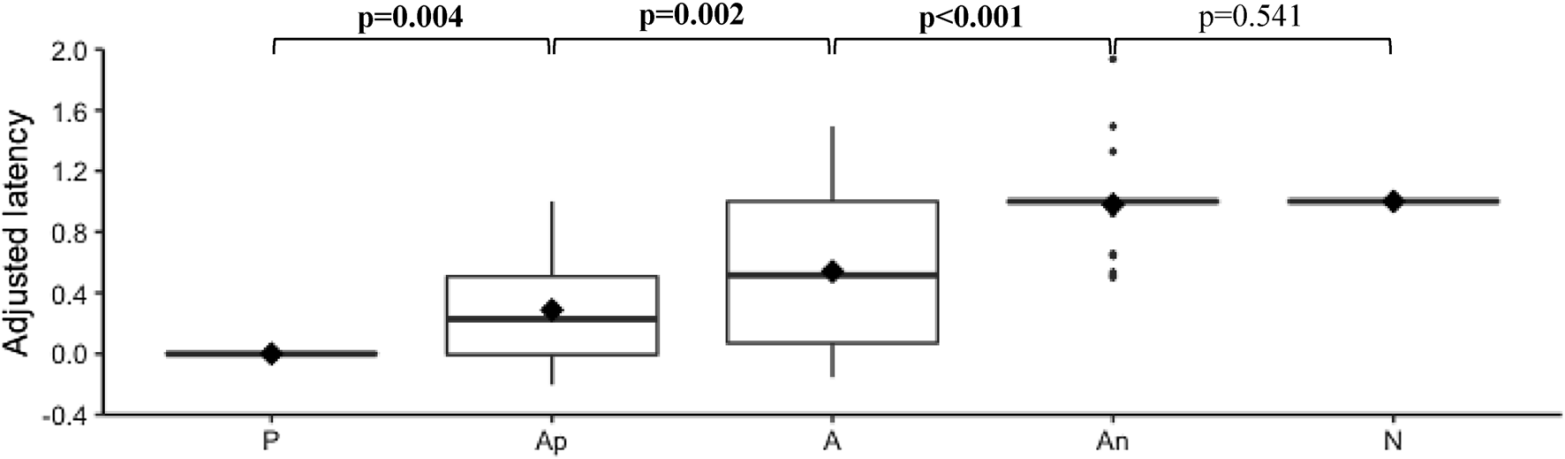
Adjusted latency according to the cue type during the first testing session. The graph represents the overall adjusted latencies obtained for all cows regardless of the punisher. For each box, ♦ represents the mean value. *P* positive cue, *Ap* ambiguous positive cue, *A* ambiguous cue, *An* ambiguous negative cue, *N* negative cue. *NOTH* punisher is the absence of a reward, *AIR* punisher is an air puff, *ELEC* punisher is an electric shock. Significant *p*-values are written in bold. R version 4.0.5 was used to create the artwork

Regardless of the punisher, cows that reached the training criterion went significantly faster to P than to Ap (V=44), to Ap than to A (V=29) and to A than to An (V=1). However, there was no statistical evidence that cows reached An significantly faster than N (V=21) Results are detailed in Fig. 7.

At the punisher level, there was no statistical evidence that NOTH-cows reached P significantly faster than A (adjusted latency to A: 0.32 ± 0.253, IQR=0.39, p=0.281, V=12) but NOTH-cows tended to reach A faster than N (*p*=0.094, V=2). AIR-cows reached A significantly slower than *P* (0.29 ± 0.072, IQR=0.42, *p*=0.004, V=0) and faster than N (*p*=0.004, V=0). ELEC-cows reached A significantly slower than P (0.91 ± 0.060, IQR=0.0, p=0.004, V=0), but there was no statistical evidence that they reached A significantly faster than N (*p*=0.371, V=0).

### Sensitivity

Within cows who met the training criterion, there was no statistical evidence for significant differences in the signed area *SA* between NOTH-cows and AIR-cows (NOTH: 0.33 ± 0.618, IQR=2.30; AIR:  – 0.20 ± 0.181, IQR=0.92 ± 0.140; *p*=0.776, W=30), but *SA* was significantly smaller for AIR-cows compared to ELEC-cows (ELEC: *SA*=0.96 ± 0.140, IQR=0.68; p<0.001, W=87). Additionally, there was no statistical evidence for significant differences in *SA* between NOTH- and ELEC-cows (*p*=0.355, W=21).

**Fig. 8:**
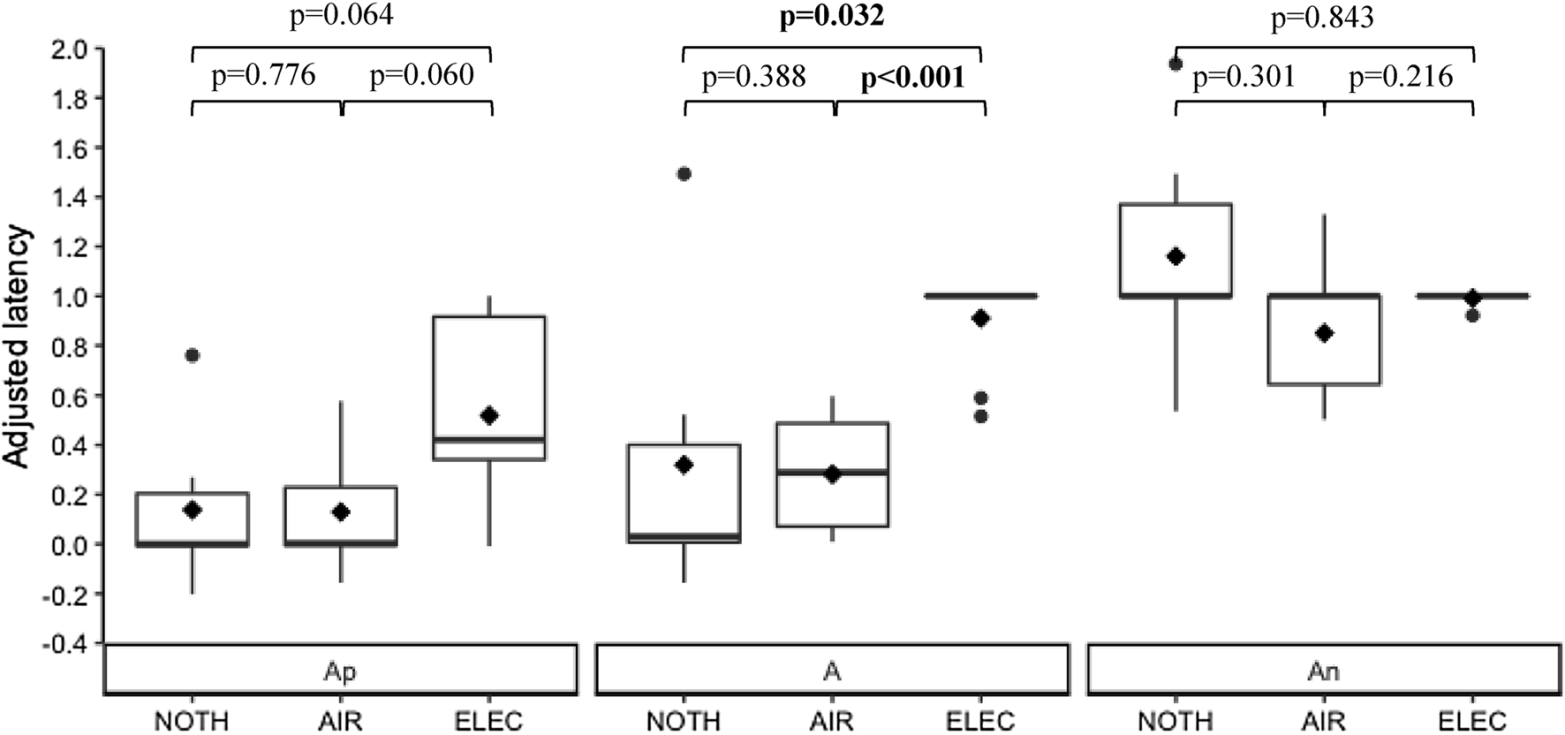
The graph depicts the box plots of the adjusted latency to the ambiguous cues according to the punisher during the first testing period. For each box, ♦ represents the mean value. *NOTH* punisher is the absence of a reward, *AIR* punisher is an air puff, *ELEC* punisher is an electric shock, *Ap* positive ambiguous cue, *A* ambiguous cue, *An* ambiguous negative cue. Significant *p*-values are written in bold. R 4.0.5 was used to create the artwork

At the ambiguous cue level, there was no statistical evidence that the adjusted latencies to Ap were significantly smaller for NOTH-cows compared with AIR-cows (W=24), but the adjusted latencies to Ap tended to be smaller for AIR-cows compared with ELEC-cows (W=21.5). Additionally, the adjusted latencies to Ap tended to be smaller for NOTH-cows compared with ELEC-cows (W=12.5). Similarly, there were no statistical evidence that the adjusted latencies to A were significantly smaller for NOTH-cows compared with AIR-cows (W=19), but the adjusted latencies to A were significantly smaller for AIR-cows compared with ELEC-cows (W=2). Similarly, the adjusted latencies to A were smaller for NOTH-cows compared to ELEC-cows (W=11). Finally, there was no statistical evidence for significant differences in adjusted latencies to An between the punishers (NOTH vs AIR: W=36, AIR vs ELEC: W=32, NOTH vs ELEC: W= 32). Results are detailed at the punisher and ambiguous cue levels in Fig. 8.

### Repeatability

**Fig. 9:**
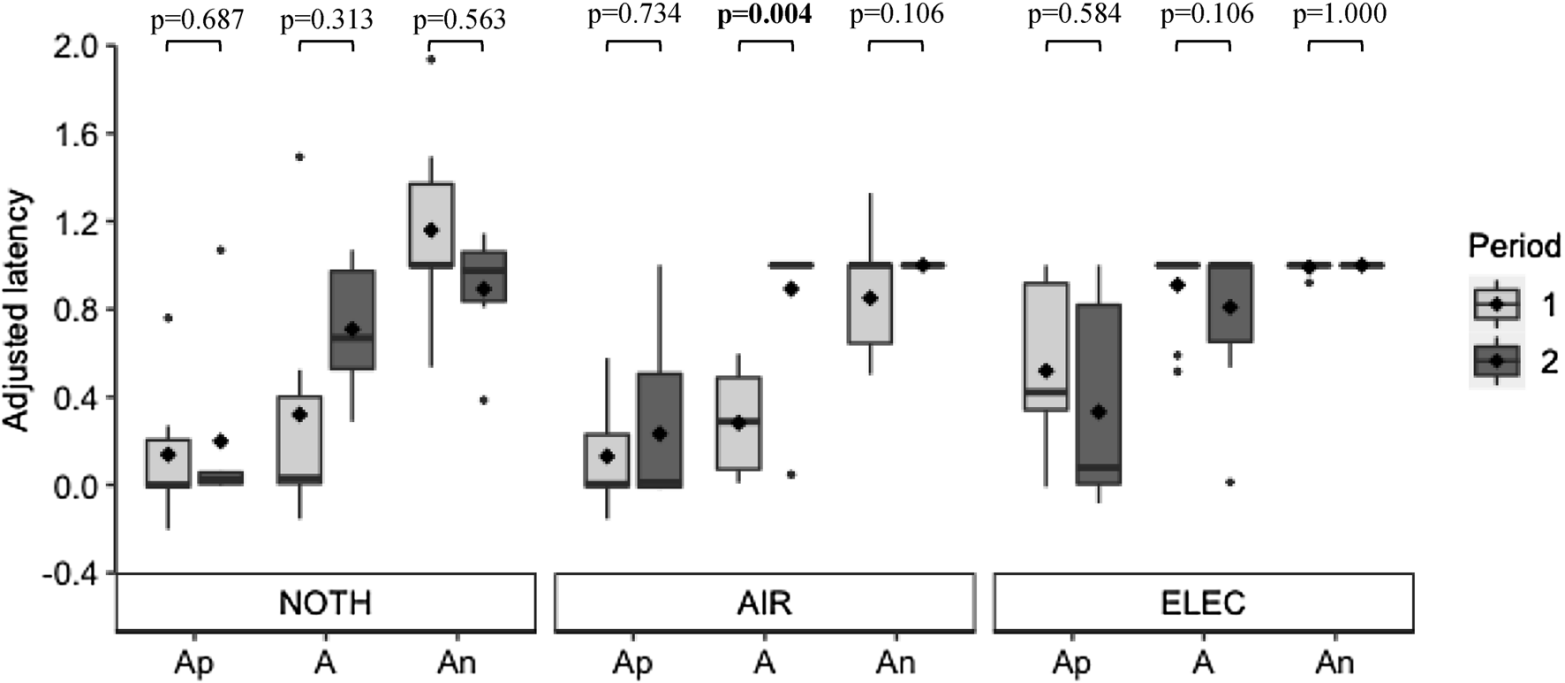
Adjusted latency to the ambiguous cues according to the punisher and the testing period. The higher the adjusted latency, the slower the cow reached the cue. For each box, ♦ represents the mean value. *NOTH* punisher is the absence of a reward, *AIR* punisher is an air puff, *ELEC* punisher is an electric shock, *Ap* ambiguous positive cue, *A* truly ambiguous cue, *An* ambiguous negative cue. Significant *p*-values are written in bold. R version 4.0.5 was used to create the artwork

Regardless of the punisher, there was no statistical evidence for significant differences in adjusted latencies to reach Ap (NOTH: V=8, AIR: V=19, ELEC: V=27) or An (NOTH: V=14, AIR: V=1, ELEC: V=0) between the first and the second testing periods. However, adjusted latencies to A were significantly higher for AIR-cows in the second testing period compared with the first (V=0), while there was no evidence for significant differences in adjusted latencies to reach A between the two testing periods for NOTH-cows (V=5) and ELEC-cows (V=7). Fig. 9 provides an overview of these results. Furthermore, there was no statistical evidence for significant correlations between adjusted latencies to the ambiguous cues in the first testing period on the one hand and adjusted latencies to the ambiguous cues in the second testing period on the other hand - when cows reached the ambiguous cues (Table 1).

**Table 1:** Spearman’s rank correlation coefficients (with respective *p*-values and S-values) between adjusted latencies during the first testing session and adjusted latencies during the second testing session for each ambiguous cue according to the punisher. Ap ambiguous positive cue, A truly ambiguous cue, An ambiguous negative cue, NOTH punisher is the absence of a reward, AIR air puff, ELEC electric shockAp ambiguous positive cue, A truly ambiguous cue

Punisher	Ap	A	An
NOTH	– 0.37 (*p* = 0.497, S = 48)	0.49 (*p* = 0.356, S = 18)	0.03 (*p* = 0.957, S = 34)
AIR	– 0.12 (*p* = 0.776, S = 134)	0.55 (*p* = 0.127, S = 54)	NA*
ELEC	0.17 (*p* = 0.643, S = 137)	0.15 (*p* = 0.681, S = 140)	NA*

^*^Coefficients with *p*-values and S-values are unavailable due to null variance in adjusted latencies to reach An for AIR-cows and ELEC-cows during the second testing period

## Discussion

The study aimed to develop for dairy cows a feasible, valid and sensitive JBT—the repetition of which does not lead to ambiguity loss. To this end, we investigated the influence of different punisher/reward combinations on the responses of cows trained and tested repeatedly in a JBT paradigm.

### Feasibility of the judgement bias tasks

The feasibility of the JBTs, here defined as cow aptitude to learn the discrimination task, was assessed based on both learning success and contingency learning. The effect of the reward/punisher combination on the JBT’s feasibility yielded mixed results. Hypothesis 1 is, therefore, only partially supported by the present study.

We did not find statistical evidence for differences in learning success between cows exposed to different combinations of reinforcers. This result suggests that all three combinations of reinforcers can be successfully used to train the majority of dairy cows on spatial discrimination relatively fast (i.e. within 56 trials in 8 sessions). This result is consistent with previous findings showing that dairy cows could successfully learn to discriminate between two conditioned spatial cues associated with either concentrates or “no-reward” (Crump et al. 2021). Former studies also demonstrated that calves were able to discriminate between two spatial cues paired either with a milk reward or an air puff (e.g. Lecorps et al. 2018).

Although learning success appeared not affected, the combination of reinforcers significantly influenced cow contingency learning (i.e. Go-to-P and NoGo-to-N). The greatest difference in responses to the conditioned cues was observed between NOTH-cows and ELEC-cows. NOTH-cows were the most likely to reach both conditioned cues and they displayed the lowest number of correct responses to N; while ELEC-cows were the least likely to reach both conditioned cues and they displayed the lowest number of correct responses to P. NOTH-cows were also faster to reach P than ELEC-cows. Additionally, AIR-cows made more correct responses to P and less correct responses to N than ELEC-cows. The “no-reward” punisher was thus associated with the worse NoGo-to-N contingency learning, while the electric shock was associated with the worse Go-to-P contingency learning. In comparison to the air puff, the “no-reward” punisher hence encourages active responses to N while the electric shock inhibits active responses to P. We, therefore, question the suitability of “no-reward” and electric shock as appropriate punishers in Go/NoGo JBTs for adult cows. Instead, we recommend using an air puff to design relatively feasible JBTs for dairy cows. There are several possible and compatible explanations for these findings.

The differences in probabilities of reaching the conditioned cues based on the punisher could arise from differences in affective responses to the punishers themselves. These punisher-induced affective states may be associated with distinct behaviours. We hypothesise that the air puff and electric shock elicited fear - an emotional state experienced in anticipation of threatening or dangerous stimuli (Papini et al. 2019). AIR- and ELEC-cows may hence display NoGo responses to N to avoid subsequent negative outcomes. Avoidance behaviours are expected in animals experiencing fear (Gray and McNaughton 2000). We hypothesise that “no-reward” instead elicited frustration—*“a temporary state that results when a response is nonreinforced […] in the presence of a reward expectancy”* (Amsel 1992). A vast behavioural repertoire has been linked to frustration, including goal-oriented behaviours such as aggressiveness (Dantzer et al. 1980) and response invigoration (Papini et al. 2019). Response invigoration is characterised by an increased motivation to engage in the dominant behaviour—here, reaching the feeder. Punishers inducing avoidance rather than goal-oriented behaviours are likely to ensure more efficient learning of the NoGo response. Response suppression (i.e. NoGo) to a punisher inducing frustration is likely to be less natural than response suppression to a punisher inducing fear or pain. Therefore, the congruence between the expected behavioural response to the conditioned cue and the punisher-driven affective state is likely to reduce the required training period, thereby leading to a more feasible Go/NoGo JBT. In this respect, species-specific differences should be considered, as the adaptive responses to fear may differ from one species to the next. For instance, mice more readily learnt the Go-to-N contingency than the NoGo-to-N contingency, while rats more readily learnt the NoGo-to-N contingency (Jones et al. 2017).

Additionally, differences in probabilities to reach the conditioned cues created by the punisher may reflect differences in speed-accuracy trade-offs made by the cow during decision-making. In sensory discrimination tasks, accuracy and speed of decision are two key conflicting factors that contribute to decision quality—decisions taken faster more likely leading to errors (Chittka et al. 2009). In our experiment, ELEC-cows may have perceived the cost of making an error as higher than NOTH-cows, which may have led them not to respond to certain P trials. As a result, decision-making in ELEC-cows may have predominantly relied on accuracy gain over speed gain, while the opposite may be true for NOTH-cows.

Discrepancies in contingency learning may also have arisen from differences in punishers’ aversiveness. We hypothesise that cows experienced the electric shock as more aversive than the air puff or “no-reward”. Manipulation of the punisher aversiveness has been found to influence the acquisition of the NoGo-to-N contingency. For instance, rats subjected to electric shocks of high intensity learn to avoid the negative cue faster than rats subjected to electric shocks of lower intensity (Feigley and Spear 1970). The validity of this theory could be investigated by assessing cows’ affective arousal during their exposure to the punishers, via analyses of heart rate or thermography data for instance (Sinha et al. 1992; Clay-Warner and Robinson 2015).

Two main elements may explain the fact that we detected an effect of the reward/punisher combination on cow learning contingency (number of correct responses) but not on learning success. First, our measure of learning speed was by definition dependent upon our training criterion. Each cow was allowed to make one incorrect response to either P or N over two consecutive sessions to be considered trained. NOTH- and ELEC-cows may, thus, have reached the training criterion in a similar timespan while predominantly displaying incorrect responses to N and P, respectively. Although our training criterion is in range with other criteria found in literature (Hintze et al. 2018; Lecorps et al. 2018), opting for a different criterion (e.g. one incorrect response to N only) may have led to more contrasted results in terms of learning speed according to the allocated punisher. Second, the limited number of cows used in our experiment may have impacted the statistical power of our test - thereby reducing our ability to spot a potential effect of the punisher on cow learning success. Replication studies at a larger scale are, therefore, required to draw more reliable conclusions on the effect of the punisher/reward combination on JBT feasibility.

**Fig. 10:**
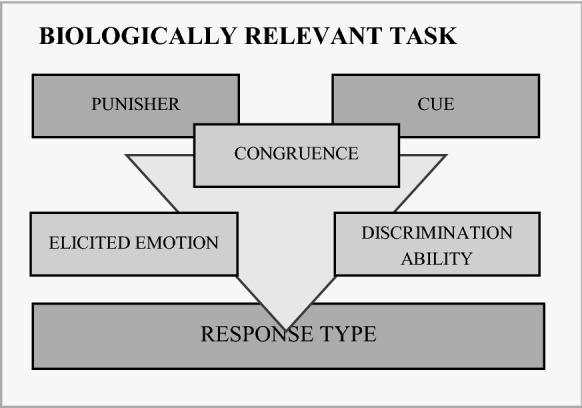
Punisher–Cue–Response triad. The rationale behind the selection of a punisher within the context of a JBT should integrate the existing congruence between the punisher and the cue modality. The chosen cue modality should be evolutionary relevant and match the species-specific discrimination ability. The punisher should elicit an emotion in line with the expected response*.* Microsoft PowerPoint was used to create the artwork

Careful consideration of factors other than the reward/punisher combination may help optimise discrimination training in JBT paradigms. The congruence between the selected punisher and the cue modality could facilitate the acquisition of the discrimination task. In rats, for instance, pairing the ingestion of a toxin with tasty water (i.e. gustatory cue) led to aversive reactions to water consumption but not pairing the ingestion of a toxin with noisy and bright water (i.e. auditory–visual cue) (Garcia and Koelling 1966). Evolutionary mechanisms may explain that internal discomfort is more readily associated with gustatory over auditory cues (Garcia and Koelling 1966). We advise that the rationale behind the choice of a punisher integrates the nature of the expected behavioural response as well as the cue modality (Fig. 10). Additionally, allowing animals to self-initiate the trials by displaying a natural behaviour (Jones et al. 2018) may also reduce the training duration. In their study, Jones et al. (2018) developed an automated JBT during which they trained rats to self-initiate a trial by nose-poking into a feed trough. Depending on the tone of a sound cue, rats learnt to leave their head in the feed trough for 2 s to get a feed-reward, or to remove their head from the trough to avoid an air puff. Compared with results obtained in other studies where rats could not initiate the trials on their own (e.g. Parker et al. 2014), Jones and colleagues reported that their rats needed fewer training sessions to be considered trained. Translation of this task to cows seems promising since dairy cows in modern commercial farms are generally used to receive concentrates in their home-pen from an automatic feeder and based on voluntary approach. Voluntary testing would also alleviate some of the feasibility issues encountered in the present study. First, habituation time would be significantly reduced since training would occur in a familiar environment with a familiar device. Second, automation of the delivery of concentrates/air puff would considerably reduce the time allocated by researchers to training. Third, self-initiation of trials would give cows control over the task which is expected to guarantee motivation to participate (Hintze et al. 2018).

### Internal validity of the Judgment Bias Tasks

The internal validity of a spatial task was assessed by investigating the pattern of cow responses (expressed in adjusted latencies) from the positive to the negative cues for all punishers. Overall, cows reached the adjacent spatial cues at significantly different speeds regardless of the punisher used, which is in line with our hypothesis 2. This finding corroborates the idea that cows are able to discriminate between two cues separated from 1.6 m, and is in agreement with another study assessing the horizontal visual acuity of Friesian-Holstein dairy bulls at 1.6 c/deg (Rehkämper et al. 2000). Furthermore, when combining all punishers, the monotonic graded pattern of responses from the positive to the negative cues was effectively observed, which indicates that cows exhibited more optimistic responses to ambiguous cues positioned closer to the positive conditioned cue, and vice versa. This result supports the idea that cows perceived the positions of the feeder between the conditioned cues as ambiguous rather than novel (Hintze et al. 2018) and further validates the use of a spatial Go/NoGo JBT in dairy cows.

**Fig. 11:**
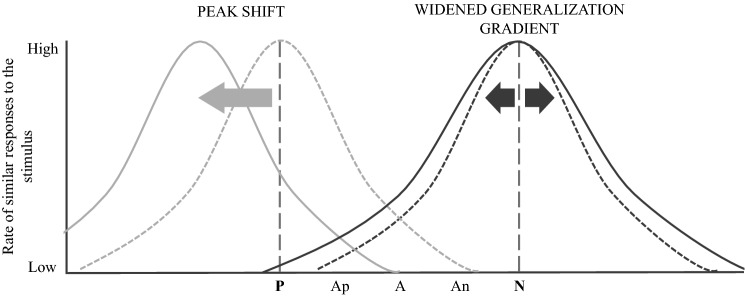
Peak shift phenomenon and generalization gradient expansion (adapted from Roelofs et al. 2016). When the negative value of N outweighs the rewarding value of *P*, the generalisation gradients around *P* may shift away from N. When the threat linked to *N* is severe, the discrimination thresholds between perceptually similar cues (for instance A, An and N) may decrease. Microsoft PowerPoint was used to create the artwork

Nonetheless, differences in adjusted latencies to reach the truly ambiguous cue A relative to P and N were noted. While NOTH-cows reached A and P at a similar speed (statistically speaking) and ELEC-cows reached A and N at a similar speed, AIR-cows reached A slower than P and faster than N. We suggest that, within each JBT task, individual responses to the ambiguous cue are partially determined by the asymmetry in the affective ladder delineated by the reinforcers (from rewarding/pleasant to aversive/unpleasant). Affective asymmetry may lead to a phenomenon known as “peak shift” (Roelofs et al. 2016) which results in biased responses to the ambiguous cues toward the most salient cue -i.e. the cue associated with the reinforcer of the highest value, be it negative or positive (Fig. 11). For NOTH-cows, the perceived positive value of the concentrates may have largely exceeded the perceived aversive value of the absence of reward, resulting in responses to the ambiguous cue biased toward P. In contrast, the aversive value of the air puff appears to balance the positive value of the concentrates, leading AIR-cows to reach A at an intermediary speed. Finally, the aversive value of the electric shock may have outweighed the rewarding value of the concentrates, leading to responses to ambiguous cues biased to N in the ELEC-cows. Above a certain intensity, cow motivation to avoid an electric shock becomes greater than feed motivation—as demonstrated by Lee et al. (2007) who successfully trained cows to stop reaching a feeding trough filled with hay by using an electric shock. Further research is required to refine our understanding of how the punisher/reward balance may influence the JBT’s internal validity, as well as its sensitivity.

### Sensitivity of the judgement bias tasks

**Fig. 12:**
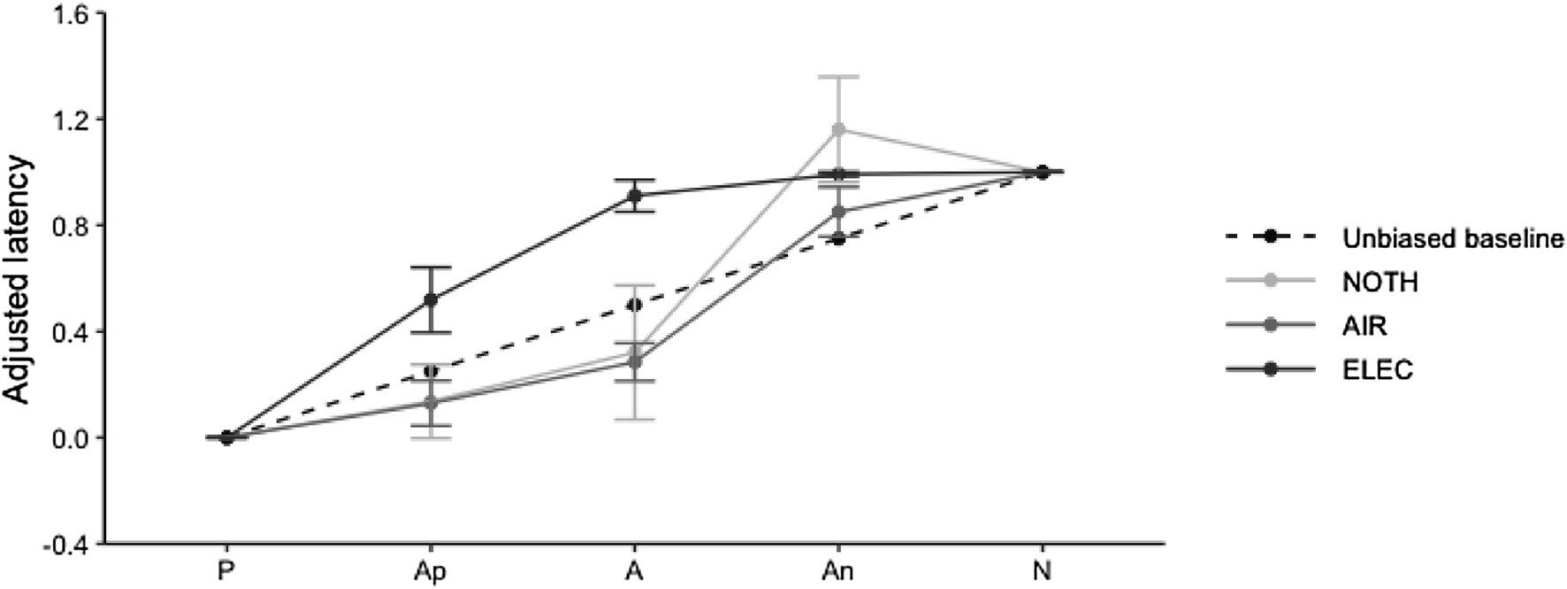
Adjusted latencies according to the cue type and the punisher for the first testing period. *NOTH* punisher is the absence of a reward, *AIR* punisher is an air puff, *ELEC* punisher is an electric shock*.* R 4.0.5 was used to create the artwork

The sensitivity of each JBT was assessed by calculating the divergence (expressed using signed area) between cow adjusted latencies to reach the cues and the expected unbiased baseline (linear and monotonic graded pattern). Divergence from this line before the application of any affective treatment reflects a construct punisher-driven bias that can mask treatment-induced judgement bias—therefore altering the sensitivity of the task. In line with our hypothesis 3, the combination of reinforcers influenced the sensitivity of the task. ELEC-cows responses to the ambiguous cues were negatively biased, while AIR-cows responses to the ambiguous cues were the closest to the expected theoretical line. In other words, ELEC-cows exhibited a negative baseline judgement bias. In our experimental design, the electric shock is therefore not associated with a sensitive JBT, and may lead to a failure to detect a treatment-induced negative affective shift. By contrast, a 5 bar air puff is a punisher suitable for a valid and sensitive JBT in cows, since the punisher-induced judgement bias was minimal for AIR-cows. Unexpectedly, NOTH-cows also exhibited an overall negatively biased baseline. The positive area between the experimental curve of NOTH-cows responses to the ambiguous cues and the theoretical unbiased baseline was, thus, larger than the negative area (as noticeable in Fig. 12). This finding can partially be explained by the fact that 3/6 trained NOTH-cows kept responding to N during testing, which resulted in adjusted latencies to An above 1. This result could also be attributed to the fact that trained NOTH-cows associated the outcomes of the unrewarded An to the outcome of N—and, thus, stopped responding to An in the same way they learnt to suppress their behavioural response to N. This result is similar to previous findings demonstrating that JBTs involving “no-reward” punishers are less sensitive than JBTs involving more salient punishers (Lagisz et al. 2020) since the “no-reward” punisher-induced judgment bias around An may hamper the detection of treatment-induced negative shifts in animal affective states. Therefore, we hypothesise that the sensitivity of such tasks could be increased by positioning An further away from N, in an attempt to reduce individual expectations of a negative outcome associated with An.

Differences in response to the ambiguous cues may have emerged from variations in cow affective states. The type of punisher may have impacted (1) cow perception of the JBT as a whole and (2) cow affective states within the JBT. First, the JBT has often been considered as a potential cognitive enrichment that could enhance animal welfare (Roelofs et al. 2016). However, although habituated, cows may still dislike being isolated and regularly handled by the experimenters. Their repeated exposure to aversive experiences when making incorrect responses to N during training may also negatively influence cow welfare - and consequently alter their motivation to engage in the judgement bias task. Thus, since cows were exposed to the training procedures up to three times per week in our experiment, we cannot rule out the possibility that the JBT training itself induced an affective shift in cows. Based on the assumption that the electric shock was more aversive than the air puff or “no-reward”, ELEC-cows may hence have been in a worse welfare state than AIR-cows and NOTH-cows, which led them to make more pessimistic decisions. Future studies are required to elucidate the influence of JBT procedure on animal welfare. This could be assessed, for instance, by investigating how motivated animals are to participate in the JBT. Behavioural and physiological differences between groups of animals exposed or not to the JBT could also be scrutinised to assess the long-term effect of JBT on animal affective states. Of note, the animal perception of the JBT is also likely to evolve, depending on how fast the individuals are able to cope with the exposure to a threat (i.e. the negative cue signalling the punisher). Over time, one might expect that the negative impact of the negative conditioned cue will decrease as the animals learn to avoid the associated punisher. The animal perception of the JBT could, thus, also improve over time, as the animals gain control over the task.

Second, even trained cows may experience negative affect inside the testing arena when faced with the negative cue that they may perceive as a threat. The (supposedly) relatively high aversiveness of the electric shock may have induced anxious-like affective states in cows, resulting in pessimistic responses to the ambiguous cues. Anxiety has been linked with negative interpretation bias, judgement bias and decision-making bias in humans (Blanchette and Richards 2010) and animals (e.g. rats: Burman et al. 2009; dogs: Karagiannis et al. 2015). Thus, compared with NOTH- and AIR-cows, ELEC-cows may have interpreted the ambiguous cues as more negative (“interpretation bias”) and overestimated the likelihood of the ambiguous cues to be associated with a negative outcome (“judgement bias”). In mechanistic terms, the supposedly higher aversiveness of the electric shock compared with the air puff or the absence of reward may have widened the generalisation gradient around N, resulting in more risk-averse decisions (decision-making bias) to the ambiguous cues. From an evolutionary perspective, it is more advantageous to react similarly to a wide range of stimuli with characteristics common to these of a stimulus associated with a severe threat (e.g. predator attack), while sharper discrimination may be more advantageous when the threat is less severe (e.g. insect bite). The influence of N on animal short-term affective states could be investigated by analysing individual behavioural indicators of affect (e.g. ear postures and vocalisations) when the latter are faced with N within the testing arena.

Factors other than the combination of reinforcers must be taken into account to design a sensitive JBT. Following the example of previous JBTs designed for herbivores, we opted for a spatial Go/NoGo discrimination tasks. However, a recent meta-analysis from Lagisz et al. (2020) revealed that the most sensitive JBTs rely on active choice tasks and involve either tactile or auditory cues. Considering the relatively wide hearing range of cows (Heffner and Heffner 1983), researchers may, thus, consider developing an auditory active choice task suitable for dairy cows rather than a spatial Go/NoGo paradigm. In active choice tasks, cows could be taught to press either a right or left panel in response to the conditioned cues - as performed by calves on a double demand operant conditioning set-up (Webb et al. 2015). Moreover, Lagisz et al. (2020) demonstrated that JBTs using reward/smaller reward as combination of reinforcers are more sensitive than JBTs using reward/punisher as reinforcers. Future studies should therefore investigate cows’ discrimination ability among different reward quantities, and subsequently determine whether using a smaller reward instead of an air puff leads to more sensitive (and potentially feasible) JBTs in dairy cows than those presented in this study.

### Repeatability of the judgement bias tasks

The repeatability of each JBT was assessed based on (1) cows’ differences in adjusted latencies to reach the ambiguous cues between the first and the second periods of testing and (2) individual consistency in response to the ambiguous cues between both testing periods. In contradiction with hypothesis 4, not all three JBTs appeared repeatable - despite the inclusion of a wash-out period between the two testing periods.

Our assessment of repeatability for the JBTs associated with “no-reward” and the electric shock was inconclusive. While there was no statistical evidence that NOTH- or ELEC-cows reached the ambiguous cues slower during the second testing period compared to the first testing period (although means did differ, hence suggesting that our sample size was too small to pick up differences), there was also no statistical support for consistency in individual responses between both periods. Non-significant results being no proof of an absence of effect, we are, here, unable to reject or confirm the hypothesis that these JBTs are repeatable. For a JBT to be characterised as repeatable, two requirements must be met—none of which is self-sufficient. First, the population study must, on average, reach the ambiguous cues in a similar timespan for every testing session. Second, each individual must be consistent in their responses to the ambiguous cues (i.e. a relatively optimistic individual should remain relatively more optimistic than the other individuals of the study population in every testing session). Nowadays, however, JBTs’ repeatability is often investigated based on the sole assessment of the first requirement. While such a strategy can be validly used to demonstrate that a task is not repeatable (Doyle et al. 2010b), it is not sufficient to demonstrate that the task *is* repeatable. In the latter case, a JBT could be falsely advertised as repeatable despite little correlation in individual responses to the ambiguous cues across testing sessions. As recommended elsewhere (Carreras et al. 2015), we, therefore, encourage researchers to assess JBT repeatability by investigating individual consistency in response to the ambiguous cues - in addition to exploring differences in response means over testing sessions.

Finally, we were able to demonstrate that the JBT associated with the air puff was not repeatable. AIR-cows reached the truly ambiguous cue slower during the second testing period compared with the first testing session. This finding could either indicate a negative shift in cow perception of the JBT between the two testing sessions or demonstrate that cows progressively learnt that the ambiguous cues were not rewarded. The second assumption seems the most plausible since our cows were subjected four times to the test, and previous studies already reported a loss of ambiguity after repeated testing (Doyle et al. 2010b; Scollo et al. 2014; Karagiannis et al. 2015). The loss of ambiguity in our study could also be explained by the low reference:ambiguous trial ratio compared with other studies (e.g. 4:3 versus 50:3 for rodents in Hintze et al. 2018). Therefore, while the air puff is associated with a relatively feasible and sensitive judgement bias task for dairy cows, we recommend reducing the number of testing sessions to minimise ambiguity loss. Our experimental design could also be combined with one of the following strategies to counteract ambiguity loss.

Partial reinforcement of the conditioned cues has been proposed to minimise ambiguity loss (Roelofs et al. 2016). In calves, Neave et al. (2013) applied a partial reinforcement ratio schedule during training - by progressively reducing the positive reinforcement by 50 %. As a result, the outcome of unrewarded ambiguous cues remained unclear to calves since the uncertainty of the reinforcement value (i.e. positive or less positive/neutral) of Go response to P was already introduced at the end of the training period. Training animals to associate ambiguous cues with pre-determined reinforcement ratio would also eliminate the risk of ambiguity loss (Lecorps et al. 2019). Lecorps et al. (2019) recently developed an innovative spatial judgement paradigm during which calves were directly trained to discriminate among the usual reference cues and three ambiguous cues. Responses to the ambiguous cues were reinforced based on the expected probability of reinforcement according to the cue position. For instance, calves were trained to associate the ambiguous cue positioned exactly in between the positive and the negative conditioned cues with a probability of getting a reward or a punisher of 50 %. Affect-driven judgement bias due to a specific treatment was therefore assessed by comparing latencies to reach the ambiguous cues before and after the treatment induction.

As much as possible, repeatable JBT procedures should be developed to assess treatment-induced affective states within longitudinal studies. Longitudinal assessment of animal judgement bias allows to control for the effect of endogenous factors – otherwise known to influence individual responses to ambiguous cues in cross-sectional studies. In future studies aiming at developing a JBT suitable for dairy cows, we, thus, suggest the use of an auditory Go/NoGo discrimination task in which cows learn the outcome of three ambiguous sound cues at the concentrate station. An air puff could be used as a punisher to ensure the acquisition of the NoGo-to-N contingency.

## Conclusion

The aim of our study was to design a relatively feasible, valid and sensitive JBT for adult dairy cows that could be used in longitudinal studies. Here, we demonstrated the validity of using spatial JBTs for dairy cows and confirmed the effect of the combination of reinforcers on JBT feasibility. Despite having no detectable effect on learning success the combination of reinforcers influenced cow contingency learning during training. Cows displayed more Go responses to both conditioned cues when using “no-reward” as a punisher, while cows displayed more NoGo responses to both cues when using an electric shock. We also demonstrated the impact of the combination of reinforcers on JBT sensitivity, and we identified the JBT associated with the air puff as the most sensitive JBT within our study. Although unfit for longitudinal studies, spatial discrimination tasks using concentrates and air puff as reinforcers constitute valid, sensitive and relatively feasible JBTs for dairy cows. Other methodological aspects, like the type of task, should be considered in the future to refine this already promising JBT for dairy cows.

## Data Availability

Datasets and scripts are available on a public repository https://doi.org/10.4121/15125193
